# A Chemiluminescence Signal Amplification Method for MicroRNA Detection: The Combination of Molecular Aptamer Beacons with Enzyme-Free Hybridization Chain Reaction

**DOI:** 10.3390/molecules29235782

**Published:** 2024-12-06

**Authors:** Yu Han, Jialin Li, Man Li, Ran An, Xu Zhang, Sheng Cai

**Affiliations:** 1 College of Pharmacy, Jilin Medical University, Jilin 132013, China; 19234368327@163.com (J.L.); 13894693730@163.com (M.L.); anran051024@163.com (R.A.); z1877620371@163.com (X.Z.); 2 Institute of Drug Metabolism and Pharmaceutical Analysis, Zhejiang Province Key Laboratory of Anti-Cancer Drug Research, Zhejiang University, Hangzhou 310058, China

**Keywords:** chemiluminescence, hybridization chain reaction, miR-146b, molecular aptamer beacon

## Abstract

The association between microRNA (miRNA) and various diseases has been established; miRNAs have the potential to be biomarkers for these diseases. Nevertheless, the challenge of correctly quantifying an miRNA arises from its low abundance and a high degree of family homology. Therefore, in the present study, we devised a chemiluminescence (CL) detection method for miRNAs, known as the hybridization chain reaction (HCR)-CL, utilizing the enzyme-free signal amplification technology of HCR. The proposed methodology obviates the need for temperature conversion and offers a straightforward procedure owing to the absence of enzymatic participation, and the lumino-H_2_O_2_-mediated CL reaction occurs at a high rate. The technique successfully detected 2.5 amol of the target analyte and 50 amol of miR-146b in a 1% concentration of human serum. In summary, the method developed in this study is characterized by its ease of operation, cost-effectiveness, remarkable analytical prowess, and ability to detect miRNA without the need for total RNA extraction from serum samples. This method is expected to be widely used for biological sample testing in clinical settings.

## 1. Introduction

MicroRNAs (miRNAs) are a category of biomarker implicated in a wide range of disorders, particularly cancer [1,2]. For instance, a correlation exists between the occurrence of cervical and ovarian malignancies and abnormal expression of miR-21 [3]. In patients diagnosed with gastric carcinoma, serum miR-let-7a levels are downregulated [4]. In contrast, the serum of individuals with papillary thyroid carcinoma has increased miR-146b-5p and decreased miR-199b-5p levels [5,6]. These studies have demonstrated the critical role of miRNAs in cancer diagnosis and treatment. However, the detection of miRNA content is hindered by several factors, mostly owing to their unique features including short sequence, high degree of similarity among sequence homology and low abundance in organisms [7]. Currently, stem-loop reverse transcription quantitative PCR (RT-qPCR) is considered the gold standard for assessing miRNA expression levels [8]. Although this method has been widely used in analytical chemistry and life sciences, it typically requires a two-step process and the sophisticated control of temperature cycling that can lead to operational challenges and high polymerase expenses [9]. Moreover, it was significant that the stem-loop RT-qPCR method had difficulty in discriminating miRNA family members with highly similar sequences. Over the past decades, isothermal amplification techniques due to their advantages, including without the precise control of temperature cycling, mild reaction conditions, and suitability for in situ detection, have emerged as an alternative method for miRNA detection [10].

At present, the principal isothermal signal amplification techniques employed for miRNA detection can be broadly categorized into two types: enzyme-free and enzyme-driven technologies. To start with, enzyme-driven isothermal signal amplification methods have carved out a significant niche in the realm of miRNA detection. Prominent examples include rolling circle amplification (RCA), loop-mediated isothermal amplification (LAMP), exponential amplification reaction (EXPAR), strand-displacement amplification (SDA), and duplex-specific nuclease signal amplification (DSNSA). These methods are renowned for their exceptionally high amplification efficiencies, which are capable of exponentially ramping up the quantity of target nucleic acids within a remarkably short span of time. This, in turn, substantially bolsters the detection limits of miRNAs, making even trace amounts more detectable. However, it is worth noting that these methods typically rely on one or two functional enzymes. DNA polymerases, in particular, are often enlisted, which unfortunately brings about the issue of non-specific amplification signals [5].

Moving on to the second category, enzyme-free isothermal signal amplification methods have emerged as a viable solution to circumvent the drawbacks associated with their enzyme-driven counterparts. Take catalytic hairpin assembly (CHA) [11] and hybridization chain reaction (HCR) [12] as illustrative cases. These processes are powered by toehold-mediated strand displacement and branch migration, which is a mechanism that obviates the need for exogenous enzymes. Moreover, their reaction conditions are mild, and they could be easily combined with other amplification technologies. This flexibility not only broadens the scope of their application but also holds great promise for future advancements in miRNA detection methodologies. Hence, the implementation of enzyme-free signal amplification techniques has the potential to streamline operational procedures as well as reduce costs and background signals [13].

The present study utilized the hybridization chain reaction (HCR) as the output terminal for signal amplification [14,15,16]. In the context of nucleic acid molecular detection, efficient signal amplification can be facilitated by creating hairpin primers (HP1 and HP2) that are specifically tailored to the target sequence (DNA or RNA). HCR signal amplification produces double-stranded DNA (dsDNA) [17]. Generally, the detection of products is achieved using fluorescent dyes and agarose gel electrophoresis [18,19]. However, these techniques exhibit limited sensitivity and specificity, rendering them unsuitable for the analysis of miRNAs with low abundance. According to the literature, the utilization of luminol-H_2_O_2_ mediated chemiluminescence (CL) has several advantages, including rapid reaction kinetics and straightforward experimental procedures [20]. Currently, the use of horseradish peroxidase (HRP)-conjugated antibodies is widespread in the field of immunology for identifying immune responses toward specific molecules present in the test sample [21,22]. H_2_O_2_ functions as a catalyst to induce photon emission from the resulting reaction product, thereby achieving a notable level of sensitivity and specificity in the analysis [23].

To facilitate CL detection, HCR hairpin primers were labeled with fluorescein isothiocyanate (FITC) molecules and subsequently bound to FITC antibodies labeled with HRP. At this point, the target signal could not be distinguished in the mixed solution. Therefore, in order to solve this problem, molecular aptamer beacons were designed [24]. The specific idea was to replace the stem base of the molecular beacon with an aptamer fragment [25,26]. Because of the presence of a secondary structure, the aptamer fragment could not specifically recognize the target molecule. When the secondary structure of the molecular beacon primer was opened, the complete aptamer fragment was exposed and could bind to the target molecule. Subsequently, CL techniques were used for signal detection.

Based on the above background information, a molecular aptamer beacon (MAB) primer containing streptavidin aptamer sequences and triggering HCR reaction sequences that can specifically recognize the target miRNA were designed [19,27]. HP1 and HP2 can be initiated by activation of the trigger chain, resulting in the occurrence of HCR, one of which is labeled with FITC. After completion of the reaction, the dsDNA products were separated using streptavidin-coated magnetic beads (SA-MBs). Subsequently, the products were incubated with HRP peroxidase-labeled antibodies. This process was undertaken to establish a novel approach for the detection of miRNAs based on the principles of HCR and chemiluminescence binding. This is referred to as the HCR-CL method. This approach enabled the swift and accurate examination of miR-146b, exhibiting exceptional sensitivity and specificity. Moreover, it effectively mitigated the impact of biological matrix effects, thus offering a robust detection methodology for the biochemical investigation of miRNAs.

## 2. Results

### 2.1. Principle of miRNA Detection

**Figure 1:**
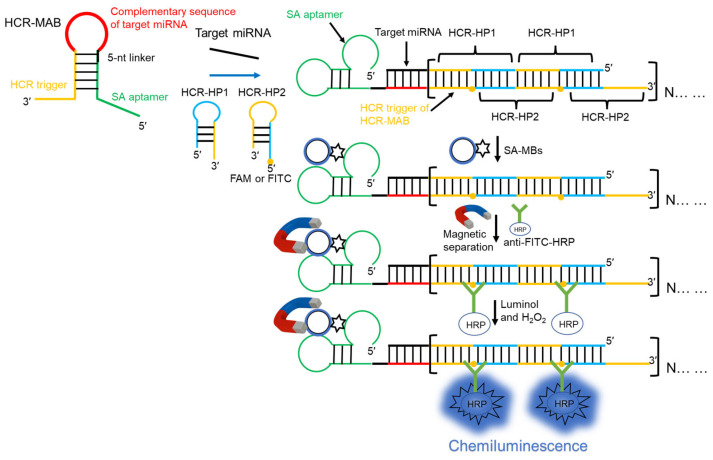
Schematic representation of the principle based on the enzyme-free hybridization chain reaction (HCR) and chemiluminescence for microRNA (miRNA) detection. The conformational change in the MAB primer triggered the HCR. Subsequently, the streptavidin aptamer of the primer was activated. With the magnetic separation, the HCR products were separated from the original reaction solution, achieving the purpose of specific detection. The MAB primer was labeled with FITC, and then anti-FITC-HRP was added to bind specifically. Under the combined influence and catalytic effect of luminol and H_2_O_2_, the target miRNA could be detected rapidly and sensitively. The red color represented the complementary sequences of the target miRNA. The yellow color represented the sequences of the HCR trigger. The green color represented the sequences of the SA aptamer.

The reaction principle is illustrated in Figure 1. The present study used a target miRNA as the analyte to verify the feasibility of the designed HCR-CL system. In the absence of the target miRNA, the secondary structure of the MAB primer occupied the streptavidin aptamer sequence at both ends of the primer and in the sequence that triggered the HCR reaction, resulting in an inability to recognize streptavidin or trigger the HCR reaction. When the target miRNA hybridized with the MAB primer, the secondary structure of the primer was opened, exposing the streptavidin aptamer and the base sequence that triggered the HCR reaction, thereby activating the streptavidin aptamer and enabling it to bind specifically to SA-MBs. Both HCR-HP1 and HCR-HP2 primers had a foothold region of six bases, which was used to identify the trigger chain and stem region of HCR-HP1, respectively. The stem base of HCR-HP2 was the same as that of the trigger chain and was then bound to HCR-HP1 repeatedly, achieving signal amplification. After initiating the HCR reaction, SA-MBs were added, which was followed by the addition of anti-FITC-HRP to the solution after magnetic separation. Before detection, luminol and H_2_O_2_ were added. The H_2_O_2_ catalyzed HRP, leading to the transformation of luminol into an excited-state substance. Therefore, the CL signals could be collected and achieved the highly sensitive detection of miRNA.

### 2.2. Verification of the Feasibility of the Detection System

**Figure 2:**
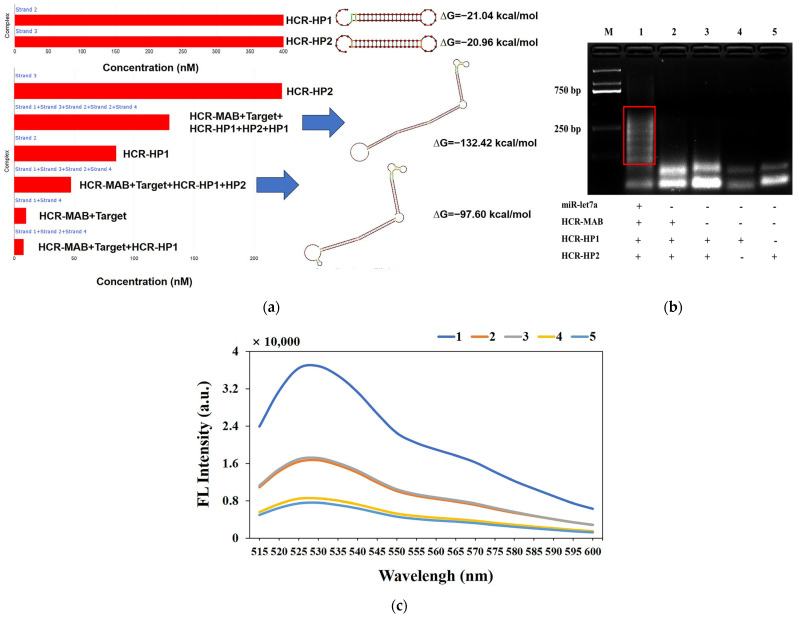
(**a**) Simulation of the HCR primers and products by NUPACK: the different colors of dots represented the equilibrium probability of hairpins primers and HCR products structures, the red box represented the concentrations of different hairpins primers and HCR products, the blue arrows represented the corresponding structure of HCR products; (**b**) agarose gel electrophoresis of HCR reaction: lane 1: miR-let7a (10 pmol) + HCR-MAB-let7a (10 pmol) + HCR-HP1 and HP2 (20 pmol), lane 2: HCR-MAB-let7a (10 pmol) + HCR-HP1 and HP2 (20 pmol), lane 3: HCR-HP1 and HP2 (20 pmol), lane 4: HCR-HP1 (20 pmol), lane 5: HCR-HP2 (20 pmol); (**c**) fluorescence spectrum of EvaGreen within the solution of HCR reaction products: 1: miR-let7a (2 pmol) + HCR-MAB-let7a (10 pmol) + HCR-HP1 and HP2 (20 pmol), 2: HCR-MAB-let7a (10 pmol) + HCR-HP1 and HP2 (20 pmol), 3: HCR-HP1 and HP2 (20 pmol), 4: HCR-HP1 (20 pmol), 5: HCR-HP2 (20 pmol).

Firstly, this HCR primers and products were simulated by NUPACK https://nupack.org/ (accessed on 1 November 2024) [28,29]; the reaction buffer and temperature were the same as in the experiment section. As shown in Figure 2a, the maximum complex product was set to be five components, and the HCR-HP1 and HP2 primers were mixed in the concentration of 400 nM. However, all primers were not binding to trigger an HCR reaction; because of that, two primers were stable. When the target and HCR-MAB primer were added, the HCR reaction was triggered and dsDNA products appeared at the reaction solution. The dsDNA products included two types of HCR products. Due to the different ΔG (Gibbs free energy) values of the two products, the reaction always tends toward a more stable direction. Therefore, in the presence of HCR-HP1 and HP2 primers, the reaction will continue, and the detection signal of the HCR reaction will be further amplified. If the maximum complex product was set to be 10 components, the more stable HCR products will continuously appear.

In addition, the agarose gel electrophoresis of HCR reaction experiment yielded the same results as our simulation. The molecular weight of lane 1 was substantially higher than that of lanes 2 and 3, indicating that in the presence of the target miRNA, the MAB primer was opened, triggering the exposure of chain base sequences, thereby activating the HCR reaction (Figure 2b). Even if MAB, HCR-HP1, and HCR-HP2 were present in the reaction solution, the triggering chain sequence was occupied and the HCR hairpin primers could not be activated. Moreover, carefully designed hairpin primers can coexist in a stable manner with each other. Therefore, there were no marked changes in the molecular weights of lanes 2, 3, 4, and 5.

The feasibility of the system was also verified using the final concentration of 1× EvaGreen. Curves 1 to 5 of the fluorescence spectrum in Figure 2c correspond to lanes 1 to 5 of Figure 2b. The fluorescence signal of lane 1 was substantially higher than that of lanes 2 and 3, indicating that when the target analyte was not present, the HP1 and HP2 primers could not be activated to trigger HCR reactions (Figure 2c). The fluorescence signals of lanes 2 and 3 showed no marked changes, indicating that the HCR reaction could not be activated when the conformation of the MAB primer was not disrupted.

Finally, agarose gel electrophoresis and fluorescent dyes were used to verify the rationality of the MAB and HCR hairpin primer design, which further demonstrated the feasibility of the miRNA detection strategy.

### 2.3. Optimization of Experimental Conditions

**Figure 3:**
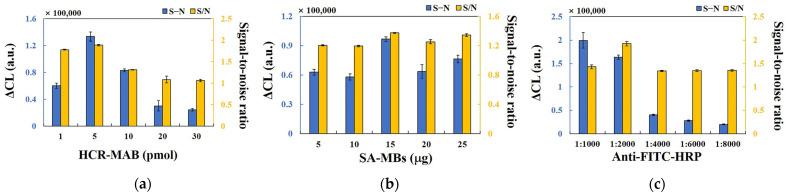
(**a**) Influence of different amounts of MAB, HCR-HP1, and HCR-HP2 on the HCR-CL system; (**b**) influence of different SA-MBs dosages on the HCR-CL system; (**c**) the different dilution ratio of anti-FITC-HRP influenced on HCR-CL. ΔCL (S − N) represents the CL intensity of the target miRNA subtracted from that of the blank. The signal-to-noise ratio (S/N) represents the ratio of CL intensity between the sample and the blank group (error bars: SD, *n* = 3).

All experimental conditions, including the amount of MAB primer, HCR-HP1, HCR-HP2, and SA-MB; and the different dilution ratios of anti-FITC-HRP were optimized (see Figure 3a–c). Briefly, the following experimental conditions were deemed optimal for efficient target miRNA detection: concentrations of MAB primer and HCR-HP1/HCR-HP2, 5 pmol and 10 pmol, respectively; the amount of SA-MB, 15 μg; the dilution ratio of anti-FITC-HRP, 1:2000.

### 2.4. Performance of Detection Strategy and Selectivity

**Figure 4:**
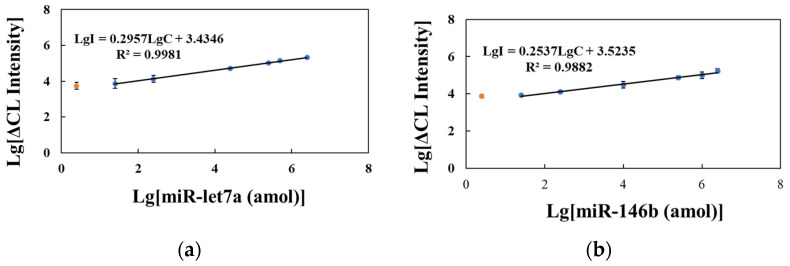
Calibration curve of the hybridization chain reaction–chemiluminescence (HCR-CL) system. (**a**) Standard solution of miR-let7a; (**b**) Standard solution of miR-146b. ΔCL represents the CL intensity of the target miRNA subtracted from that of the blank (error bars: SD, *n* = 3). The orange dots represent the minimum detected amount of the target miRNA.

Under the optimal reaction conditions, a linear regression curve using standards of different concentrations of miR-146b and miR-let7a (25 amol–2.5 pmol) was determined, where ΔCL represents the difference between the CL intensity of the sample and blank solutions with the reaction solution without the addition of target miRNA as the blank group. As shown in Figure 4a,b, the ΔCL increase in the HCR-CL system with the increase in target miRNA content had a lower limit of quantification of 25 amol (final concentration of 250 fmol/L) and a minimum detected amount of 2.5 amol (final concentration of 25 fmol/L). The linear regression equations for miR-let7a and miR-146b were LgI = 0.2957LgC + 3.4346, R^2^ = 0.9981, and LgI = 0.2537LgC + 3.5235, R^2^ = 0.9882, where I is the CL intensity and C is the target miRNA concentration.

Table S1 summarizes the relevant literature published in recent years on the use of HCR signal amplification biosensors for detecting nucleic acids (RNA or DNA) [30,31,32,33,34,35,36,37]. Compared with the analysis methods in Table S1, the HCR-CL system designed in the current study exhibited strong analytical performance. Furthermore, Table S1 also shows the detection proficiency of stem-loop RT-qPCR for the standard of miR-146b (Figure S1). This approach exhibits a detection capacity that is on a par with that of the method developed in the present study. Even without the involvement of biological enzymes, the minimum content required for the detection of miRNA is 2.5 amol, and the system demonstrates high efficiency, simplicity, and good application prospects.

**Figure 5:**
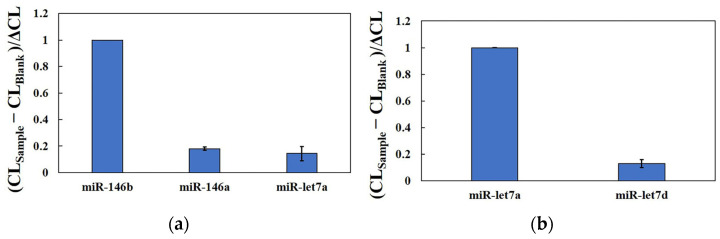
Selectivity of the HCR-CL system in different families, homologous miR-146, and miR-let detection. (**a**) The CL intensity utilized an MAB-146b probe for miR-146a and miR-let7a detection; (**b**) the CL intensity utilized the MAB-let7a probe for miR-let7d detection. CL_Sample_ represents the CL signal of miRNA standard solutions other than miR-146b and miR-let7a detected by the system; CL_Blank_ represents the CL signal of the system detecting DEPC water. ΔCL represents the CL intensity of the target miRNA subtracted from that of the blank (error bars: SD, *n* = 3).

The selectivity of the HCR-CL system for detecting the same family (miR-146a and miR-let7d) and other miRNAs was investigated in this study (Figure 5). Although miR-146a has two base mismatches with the same family as miR-146b, its CL intensity is only 18.09% of that of miR-146b. Using MAB primers to detect miR-146b, the CL intensity of miR-let7a was 14.44% of miR-146b. Using MAB primers to detect miR-let7a, the selectivity of miR-let7d as a homologous family was examined, and the CL intensity accounted for 13.05% of miR-let7a (Figure 5b). These data indicate that the newly developed strategy has excellent specificity for detecting homologous miRNA families.

### 2.5. Testing of Actual Samples

**Figure 6:**
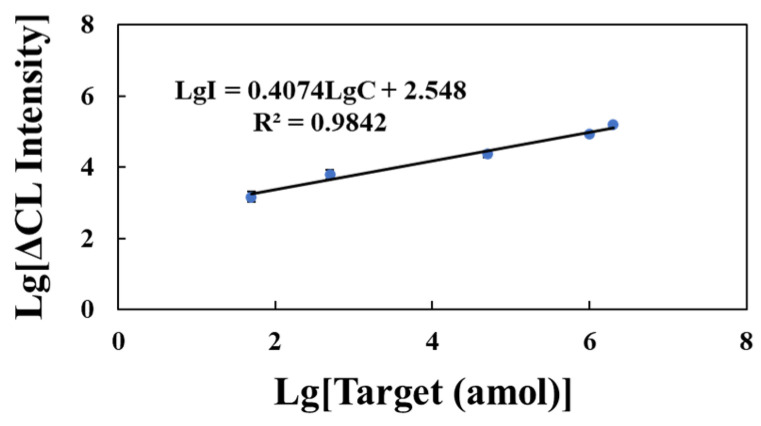
A calibration curve of miR-146b was prepared using 1% human serum (error bars: SD, *n* = 3).

To evaluate the applicability of this system for clinical disease diagnosis, human serum was extracted without using a reagent kit and diluted to 1% with a reaction buffer. Different concentrations of the miR-146b standard solution (50 amol–2 pmol) were added to the diluted serum samples. The x-axis represents the logarithm of the miR-146b standard solution in a 1% serum matrix with different substance masses, and the y-axis is the logarithm of ΔCL, where ΔCL represents the difference in CL intensity between the sample group and the blank group with serum solution without target miRNA as the blank group (Figure 6). The logarithm of ΔCL showed a positive correlation with the logarithm of miR-146b standard substance mass in the serum matrix. The linear regression equation was LgI = 0.4074LgC + 2.548, R^2^ = 0.9842.

The HCR-CL strategy developed in this study could directly detect miR-146b in serum and had good resistance to the serum biological matrix, thus eliminating the need to extract total RNA using a reagent kit, saving cost and time, and laying the foundation for the accurate analysis of miR-146b content in human blood.

The total RNA of TPC-1 cells was diluted with DEPC water to obtain a test solution of 400 ng, and different concentrations of the miR-146b standard solution (0.25, 0.5, and 1 pmol) were added to the test solution to determine the CL strength of the sample and blank groups (Table 1). A standard curve was constructed using the miR-146b standard solution. The difference in CL strength between the added sample and the blank matrix sample was substituted into the standard curve, divided by the content of the miR-146b standard, and multiplied by 100 to obtain the recovery percentage. The sample recovery rates of the system were 98.10–111.87%, and the RSD was 8.49–12.45%.

## 3. Discussion

Based on the result analysis in the previous section, the developed method presents high sensitivity and good specificity. Compared with the gold standard for miRNA detection (stem-loop RT-qPCR), although our method has a relatively higher minimum detectable concentration (2.5 amol), it features simple operation and does not require an expensive real-time qPCR instrument. Significantly, it can achieve excellent detection results and specificity without relying on enzyme use in the signal amplification stage.

Currently, miRNA detection focuses more on clinical sample detection (tissue and serum), especially for miRNAs in serum. However, the stem-loop RT-qPCR method demands complex pretreatment for the miRNA detection of serum [10]. As shown in Figure 6, our developed method overcomes serum matrix interference and skips complex pretreatment, endowing it with great prospects in early diagnosis and prognosis monitoring.

## 4. Materials and Methods

### 4.1. Reagents and Apparatus

SA-MBs (5 mg/mL, 1 μm) were purchased from Polysciences, Inc. (Warrington, PA, USA). The antibody of fluorescein isothiocyanate isomer conjugated horseradish peroxidase (anti-FITC-HRP) was purchased from Takara Biomedical Technology Co., Ltd. (Beijing, China). Immobilon Western HRP substrate, including a solution of luminol and H_2_O_2_, was purchased from Millipore. The washing buffer (WB, pH 8.0) consisted of 7 mM Tris-HCl, 130 mM NaCl, and 0.05% Tween-20. The hybridization buffer (BA, pH 8.0) contained 20 mM Tris-HCl, 500 mM NaCl, and 20 mM Mg_2_SO_4_. All DNA hairpins and probes were synthesized and purified by Sangon Biotech Co., Ltd. (Shanghai, China). Shanghai GenePharma Co., Ltd. (Shanghai, China) synthesized all miRNAs. This study employed DNA hairpins and target miRNAs, such as HCR-MAB-let7a (80 mer), HCR-MAB-146b (81 mer), HCR-HP1 (48 mer), HCR-HP2 (48 mer), miR-let7a (22 nucleotides), miR-let7d (21 nucleotides), miR-146a (22 nucleotides), and miR-146b (23 nucleotides). The base sequences of these DNA hairpins and target miRNAs are shown in Table S2.

The Nanodrop 2000 (Thermo Fisher Scientific Inc., Waltham, MA, USA) was used to measure all common DNA oligonucleotides and miRNA solutions. The primary use of the Synergy H1 multifunctional microplate detector (Berten Instruments Co., Ltd., Rockville, MD, USA) was the detection of chemiluminescence and fluorescence signals.

### 4.2. Detection of Target miRNA

Prior to the HCR, the MAB primer and two hairpin primers (HCR-HP1 and HCR-HP2) were diluted with BA to a concentration of 10 μmol/L. Subsequently, these diluted primers were subjected to a temperature of 95 °C for a duration of 5 min using the T100 Thermal Cycler (Bio-Rad Laboratories Co., Ltd., Shanghai, China). Following this, the primers were cooled down to 25 °C at a rate of 0.1 °C/s over a period of 30 min while maintaining the primers at room temperature. The HCR reaction system, with a volume of 100 μL, comprised 5 pmol of MAB primers, 10 pmol of HCR-HP1 primers, 10 pmol of HCR-HP2 primers, and varying quantities of target miRNAs. This HCR reaction was performed on a THZ type of constant temperature oscillator (Shanghai Jing Hong Laboratory Instrument Co., Ltd., Shanghai, China) at a temperature of 37 °C for 60 min. Following the completion of the reaction, 15 μL of SA-MBs was introduced and allowed to react for 60 min at 37 °C. Magnetic separation was followed by three consecutive wash cycles with 150 μL of WB solution. Subsequently, a solution containing 100 μL of 10% BSA-BA was added, and the mixture was sealed at 37 °C for 60 min.

Following the subsequent round of magnetic separation, 100 μL of anti-FITC-HRP was added at a dilution of 1:2000 and incubated for 60 min at 37 °C. The supernatant was extracted and washed three times with 150 μL of WB solution. The resulting magnetic beads were then resuspended in 50 μL of luminol and transferred to a transparent enzyme label.

Prior to conducting the experiment, it was necessary to introduce an equivalent amount of H_2_O_2_, swiftly combine the components, and promptly transfer the mixture into Synergy H1 to observe and measure alterations in CL intensity. Peak CL intensity was chosen as the fundamental criterion for the optimization of reaction conditions and quantification of miRNA.

### 4.3. Agarose Gel Electrophoresis Analysis

A mixed solution consisting of 10 pmol of miR-let7a, 10 pmol of MAB-let7a primers, and 20 pmol of HCR-HP1 and HCR-HP2 primers was prepared following the experimental procedure outlined in Section 4.2 of the Materials and Methods section. First, 10 μL of the sample solution (containing 9 μL of the reaction solution and 1 μL of 10× Loading Buffer) and the loading solution were added to the prepared 2% agarose gel. The power was turned on, and the voltage was adjusted to 140 V. When the blue band moved 2/3 of the gel, electrophoresis was stopped, and the gel was analyzed using the Bio-Rad gel imaging system.

### 4.4. Treatment of Healthy Human Blood and the Collection of Total RNA Samples from Cells

Blood from a healthy human patient (30 years old, male) was diluted with BA to 1% for subsequent use. Standard solutions of 1, 0.5, and 0.25 pmol miR-146b were added to the total RNA solution of thyroid cell line cells extracted using the reagent kit. The experimental method was described in Section 4.2 of the Materials and Methods section. The difference in CL intensity was incorporated into the standard curve equation and the content of miR-146b in the sample was calculated using the equation: recovery rate (%) = (sample content/actual sample content) × 100. All experiments were performed in accordance with the guidelines of the Declaration of Helsinki and were approved by the Ethics Committee (No.: 2024031505) of Jilin Medical University Affiliated Hospital.

## 5. Conclusions

This study used SA-MBs as a tool for separating HCR products and developed a new method for miRNA detection based on molecular aptamer beacons (MABs) and HCR signal amplification. The use of MABs to block streptavidin aptamers and trigger the base sequence of HCR substantially reduced background signal leakage, thereby achieving the specific detection of miRNAs and their family sequences. To further amplify the detection signal, anti-FITC-HRP was introduced, and luminol and H_2_O_2_ were selected as detection reagents to achieve highly sensitive analyte detection (2.5 amol). The entire detection process was rapid and did not require temperature changes. The new methods developed in this study for the HCR- and chemiluminescence-based enzyme-free detection of miRNAs were used to investigate their matrix effects in human serum and TPC-1 cells. This not only provides novel ideas for the detection of miRNAs as tumor biomarkers in human serum but also exposes new avenues for the development of chemiluminescence detection technology for HCR aptamers.

## Figures and Tables

**Table 1:** Recovery of miR-146b spiked into total RNA of TPC-1 cell (*n* = 3).

Added (fmol)	Found (fmol)	Recovery (%)	RSD (%)	Mean Recovery (%)
1000	981.00 ± 83.27	98.10	8.49	103.32
500	559.33 ± 52.34	111.87	9.36	
250	249.99 ± 31.13	99.99	12.45	

## Data Availability

Data are contained within the article and Supplementary Materials.

## Supplementary Materials

The following supporting information can be downloaded at https://www.mdpi.com/article/10.3390/molecules29235782/s1, Figure S1: The standard curve of miRNA-146b detected by stem-loop RT-qPCR. Table S1: Comparing the analytical performance of the novel developed HCR-CL with that of other HCR methods; Table S2: Sequences of DNA and miRNA used in the study (5′-3′).
