# Supplementary material for: A Chemiluminescence Signal Amplification Method for MicroRNA Detection: The Combination of Molecular Aptamer Beacons with Enzyme-Free Hybridization Chain Reaction
Source: Molecules. 2024 Dec 6;29(23):5782. doi: 10.3390/molecules29235782 (PMC11643668; doi:10.3390/molecules29235782)
Supplement: Supplementary file 1 [file molecules-29-05782-s001.zip › molecules-3322456-supplementary.pdf]

Supporting Materials

A Chemiluminescence Signal Amplification Method for MicroRNA Detection: The Combination of Molecular Aptamer Beacons with Enzyme-free Hybridization Chain Reaction

Yu Han [1*], Jialin Li [1], Man Li [1], Ran An [1], Xu Zhang [1], Sheng Cai [2*]

[1] College of Pharmacy, Jilin Medical University, Jilin 132013, China

[2] Institute of Drug Metabolism and Pharmaceutical Analysis, Zhejiang Province Key Laboratory of Anti-Cancer Drug Research, Zhejiang University, Hangzhou, Zhejiang 310058, China

* Corresponding author, E-mail: hanyu.jlmu@vip.163.com; caisheng@zju.edu.cn.

Experimental methods

Cell culture

The cells were cultured in DMEM medium supplemented with 10% fetal bovine serum, 100 U/mL penicillin, and 100 μg/mL streptomycin under a cell culture incubator set at 37°C with a 5% CO_2 atmosphere and appropriate humidity to ensure optimal growth and viability.

Extraction of total RNA from human thyroid cancer cells (TPC-1)

The human thyroid cancer cells (papillary) TPC-1 used in this experiment were purchased from Procell (Wuhan, China). The total RNA of the cells was extracted using the AxyPrep Total RNA Miniprep Kit. The specific procedures were as follows: The medium in the culture dish was removed, and 300 μL of cell lysis buffer (Buffer R-Ⅰ) was added. The cells were then detached by pipetting. Subsequently, 110 μL of neutralization buffer (Buffer R-Ⅱ) was added. After mixing, the mixture was centrifuged at 12,000 × g for 5 minutes, and the supernatant was taken and mixed with 200 μL of isopropanol by vortexing. The above solution was transferred to a preparation tube and centrifuged at 6,000 × g for 1 minute. After removing the filtrate, 500 μL of washing buffer (Buffer W1A) was added, and the tube was centrifuged at 12,000 × g for 1 minute. After removing the filtrate, 700 μL of desalting buffer (Buffer W2) was added, and the tube was centrifuged at 12,000 × g for 1 minute. This step was repeated twice. Finally, 100 μL of DEPC water was added. After standing at room temperature for 1 minute, the tube was centrifuged at 12,000 × g for 1 minute to obtain the total cell RNA solution. The concentration of the total RNA was measured using a Nanodrop. An A260/A280 ratio of 2.0 indicated successful extraction of the total cell RNA. The extracted total RNA solution was stored in a -80°C freezer.

Processing of whole blood from healthy volunteer

Whole blood was drawn by the method of venous blood collection. Vacuum blood collection tubes without any anticoagulants were used, and usually about 2 mL of blood was collected. In the laboratory, the whole blood samples were usually placed in an environment at around 37 °C for a certain period of time to allow the blood to clot naturally. After that, centrifugation was

carried out. The centrifugation speed was around 4,000 rpm, and the centrifugation time was 30 minutes, so as to separate the serum from the blood cells. The clear yellow liquid on the upper layer was the serum. After collection, it was stored in an environment at -80°C.

Detection of the content of miR-146b in PTC cell by stem-loop RT-qPCR

Firstly, the target miRNA needs to be transcribed into cDNA: A 20 μL reaction solution contains the total RNA sample, stem-loop primers, 1× RT Mix and HiScript II enzyme mixture for the synthesis of the cDNA strand. Place the above mixed solution into a PCR instrument, and set the program as follows: maintain at 25°C for 5 minutes; maintain at 50°C for 15 minutes; maintain at 85°C for 5 minutes. Subsequently, use the synthesized cDNA as a template for qPCR detection. The qPCR detection system includes 2× miRNA Universal SYBR qPCR Master mix, forward primers (10 μmol/L), reverse primers (10 μmol/L), template DNA/cDNA and ddH_2O. The qPCR detection program is as follows: After pre-denaturation at 95° C for 30 seconds, conduct 40 cycles of 95°C for 5 seconds and 55°C for 30 seconds.

The stem-loop RT-qPCR used primers following sentence. Stem-loop primer: GTCGTATCCAGTGCAGGGTCCGAGGTATTCGCACTGGATACGACCAGCCT, Forward primer: CGCGTGAGAACTGAATTCCAT, Reverse primer: AGTGCAGGGTCCGAGGTATT.

Table S1. Comparing the analytical performance of the novel developed HCR-CL with that of other HCR methods

Target analyte	Detection method	Detection range	Limit of detection	Refs
miR-21<br>miR-203	Fluorescence	0.1–1 nmol/L	1.4 or 2 pmol/L	[30]
miR-21	Dual electrochemical	0.005–10 pmol/L	3.74 fmol/L	[31]
miR-21	Fluorescence	0.005–100 nmol/L	3.3 pmol/L<br>(165 amol)	[32]
miR-17	Photoelectrochemical	0.000001–10 μmol/L	1 pmol/L	[33]
miR-21	Silver nanoclusters	0.25–8 nmol/L	19.9 pmol/L	[34]

miR-let7a	Fluorescence	0.01–10 nmol/L	2.5 pmol/L	[35]
DNA	Fluorescence	5–72.7 nmol/L	2 nmol/L	[36]
DNA	Electrochemical signal	0.00001–10 μmol/L	1 pmol/L	[37]
miR-146b miR-let7a	Chemiluminescence	0.00025–25 nmol/L	25 fmol/L (2.5 amol)	This method
miR-146b	Stem-loop RT-qPCR	0.0005–50 nmol/L	250 fmol/L (1 amol)	The data obtained in this study.

Table S2. Sequences of DNA and miRNA used in the study (5′-3′)

Name	Sequences (5′-3′)
HCR-MAB-let7a [a, c]	**ATTGACCGCTGTGTGACGCAACACTCAATAACTATACAACCT ACTACCTCA**GTTATT**GAGTGTTACTAGGATTCGGCGTG**
HCR-MAB-146b [a, c]	**ATTGACCGCTGTGTGACGCAACACTCAATCAGCCTATGGAAT TCAGTTCTCA**CTGATT**GAGTGTTACTAGGATTCGGCGTG**
HCR-HP1 [a]	**TTACTAGGATTCGGCGTGGGTTAACACGCCGAATCCTAGTAA CACTCA**
HCR-HP2 [a]	**TTAACCCACGCCGAATCCTAGTAATGAGTGTTA CTAGGATTCGGCGTG**
HCR-HP2-FITC [a]	FITC-**TTAACCCACGCCGAATCCTAGTAATGAGTGT TACTAGGATTCGGCGTG**
miR-146a [b]	UGAGAACUGAAUUCCAU***G***GG***U***U
miR-146b	UGAGAACUGAAUUCCAUAGGCUG
miR-let7a	UGAGGUAGUAGGUUGUAUAGUU
miR-let7d [b]	***A***GAGGUAGUAGGUUG***C***AUAGU

a: The colors of oligonucleotide sequences correspond to those of the domains depicted in Figure 1.

b: The bases differing from those in miR-146b and miR-let7a are marked in red italic.

c: The underlined sequences represent the conserved nucleotides.

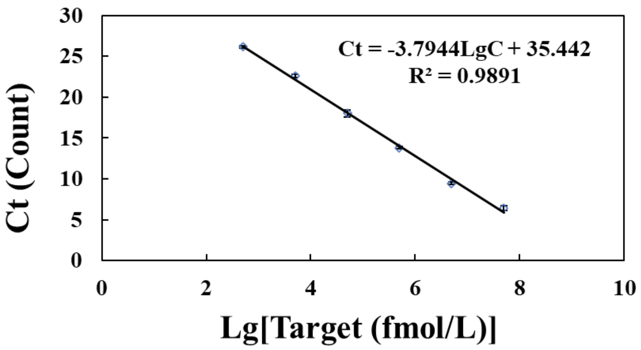

Figure S1. The standard curve of miRNA-146b detected by stem-loop RT-qPCR. (Error bars: SD, *n* = 3).

Reference:

[30] Mo, L.T.; Liang, D.L.; Mo, M.X.; Yang, C.; Lin W.Y. Dual-detection of miRNAs in living cells via hybridization chain reaction on DNA tetrahedron. *Sens. Actuator B-Chem.* **2023**, *375,* 132955.

[31] Wang, Y.H.; Feng, H.; Huang, K.; Quan, J.F.; Liu, X.H.; Jiang, H.; Wang X.M. Target-triggered hybridization chain reaction for ultrasensitive dual-signal miRNA detection. *Biosens. Bioelectron.* **2022**, *215*, 114572.

[32] Zhang, Y.F.; Chen, J.; Yang, H.H.; Yin, W.; Li, C.R.; Xu, Y.Z.; Liu, S.Y.; Dai, Z.; Zou, X.Y. Light-controlled recruitable hybridization chain reaction on exosome vehicles for highly sensitive microRNA imaging in living cells. *Anal. Chem.* **2022**, *94*, 9665–9673.

[33] Gao, G.; Hu, J.; Li, Z.; Xu, Q.; Wang, C.S.; Jia, H.M.; Zhou, H.; Lin, P.; Zhao, W.W. Hybridization chain reaction for regulating surface capacitance of organic photoelectrochemical transistor toward sensitive miRNA detection. *Biosens. Bioelectron.* **2022**, *209*, 114224.

[34] Zhang, L.; Pan, M.; Zou, Z.Q.; Fan, L.; Liu, X.Q. Hybridization chain reaction-mediated luminescent silver nanocluster system for amplified detection of miRNA-21. *Chin. J. Anal. Chem.* **2020**, *48*, 1193–1201.

[35] Ding, L.H.; Liu, H.Y.; Zhang, L.N.; Li, L.; Yu, J.H. Label-free detection of microRNA based on the fluorescence quenching of silicon nanoparticles induced by

catalyzed hairpin assembly coupled with hybridization chain reaction. *Sens. Actuator B-Chem.* **2018**, *254*, 370–376.

[36] Xu, J.; Lin, B.; Wang, Y.; Xu, Z.A.; Cheng, G.Y.; Zhang, W. Label-free fluorescent DNA detection strategy based on hybridization chain reaction and vacant site-binding molecule. *Chin. J. Anal. Chem.* **2018**, *46*, 1095–1101.

[37] Zhang, Z.K.; Zhang, L.; Liu, Y.M.; Hu, C.X.; Liu. Q.J. Sensitive DNA detection using a branched DNA as a sensor coupled with hybridization chain reaction. *ChemistrySelect* **2023**, *7*, e202201891.
